# ISMB 2024 Proceedings

**DOI:** 10.1093/bioinformatics/btae286

**Published:** 2024-06-28

**Authors:** Tijana Milenković, Yann Ponty

**Affiliations:** Department of Computer Science and Engineering, University of Notre Dame, Notre Dame, IN 46556, United States; LIX (Department of Computer Science; CNRS UMR 7161), Ecole Polytechnique, Institut Polytechnique de Paris, 91128 Palaiseau, France

## Abstract

This editorial describes the selection process and the collection of articles selected for presentation for the Proceedings Track of the ISMB 2024 conference.

This special issue of *Bioinformatics* serves as the Proceedings of the 32nd annual conference on Intelligent Systems for Molecular Biology (ISMB). This conference took place on 12–16 July 2024, in Montréal, Canada. ISMB is the leading international forum for presenting new research results, disseminating methods and techniques, and facilitating discussions among leading researchers, practitioners and students in the field. In addition, ISMB is the flagship conference of the International Society for Computational Biology (ISCB), and the largest event of the computational biology community. In order to promote diversity, equity, and inclusion by allowing access to those unable to attend in person, the meeting was run as a hybrid conference, with online participants from around the world complementing the on-site participants.

The 58 papers published in this special issue were selected from 318 submitted full-length papers featuring original research. The submitted papers were thoroughly reviewed with each paper receiving 4.38 reviews on average, for a total of 1394 reviewer reports. For the review purpose, the submitted manuscripts were assigned to one of 11 scientific areas (see Table 2) according to authors’ preferences and research topics, allowing for minor adjustments, e.g. to avoid conflicts of interest. A special area named *General Computational Biology* was intended for submissions on emerging topics or for those manuscripts that did not fit well in other reviewing areas. In addition to selecting a preferred area, the authors could also indicate up to three preferred Communities of Special Interest (COSIs; see Table 1) that would provide the best forum for presentation of their paper. The selected papers covered a broad spectrum of topics: they were distributed across 10 areas (see Table 2) and ended up being assigned to 17 COSIs (see Table 1).

**Table 1.** COSI distribution of the accepted ISMB 2024 Proceedings papers, based on the preferences of both authors and COSI organizers.

COSI	Number of papers
3DSig	3
Bioontologies	2
BioVis	1
CAMDA	1
CompMS	3
Education	2
EvolCompGen	4
General Comp Bio	4
HitSeq	7
iRNA	2
Microbiome	4
MLCSB	9
NetBio	4
RegSys	5
Text Mining	3
TransMed	3
VarI	1

**Table 2.** Thematic areas of ISMB 2024 Proceedings submissions.^a^

Area	Area chairs	No. of Sub.	No. of Acc.	Acc.%
Bioinformatics Education and Citizen Science	Nicola Mulder, University of Cape Town, South Africa Jérôme Waldispühl, McGill University, Canada	4	2	50%
Bioinformatics of Microbes and Microbiomes	Mahendra Mariadassou, Inrae Jouy-en-Josas, France Mihai Pop, University of Maryland, USA	20	4	20%
Biomedical Informatics	Karsten Borgwardt, Max Planck Institute of Biochemistry, Germany Giulio Caravagna, University of Trieste, Italy Marinka Zitnik, Harvard University, USA	60	12	20%
Evolutionary, Comparative, and Population Genomics	Erin Molloy, University of Maryland, USA Céline Scornavacca, CNRS/Université de Montpellier, France	20	4	20%
Equity and Diversity in Computational Biology Research	Casey Greene, University of Colorado, USA Alejandra Medina Rivera, Universidad Nacional Autónoma de México	2	1	50%
Genome Privacy and Security	Gamze Gursoy, Columbia University/New York Genome Center, USA Kana Shimizu, Waseda University, Japan	1	0	0%
Genome Sequence Analysis	Christina Boucher, University of Florida, USA Rayan Chikhi, CNRS/Institut Pasteur, France	32	7	22%
Macromolecular Sequence, Structure, and Function	Jianlin Cheng, University of Missouri, USA David H Mathews, University of Rochester, USA	55	10	18%
Regulatory and Functional Genomics	Sara Mostafavi, University of Washington, USA Marcel Schulz, University of Frankfurt, Germany	57	7	12%
Systems Biology and Networks	Natasa Przulj, Barcelona Supercomputing Center, Spain Bo Wang, University of Toronto, Canada	43	6	14%
General Computational Biology	Gary Bader, University of Toronto, Canada Iman Hajirasouliha, Cornell University, USA	24	5	21%

a For each area, the table lists the Area Chairs, the number of reviewed papers, the number of accepted papers, and the acceptance rate. Note that the *General Computational Biology* area was intended for submissions on emerging topics or for those manuscripts that did not fit well in the other areas.

The reviewing of the submissions was overseen by the Senior Program Committee (SPC), which included the Proceedings Chairs (this editorial’s authors) and Area Chairs (ACs, listed in Table 2). ACs were nominated by COSIs or by the ISMB Steering Committee. Members of the SPC were responsible for recruiting the Program Committee. Reviewers (Program Committee Members and subreviewers) judged the papers based on the novelty of computational approaches, relevance of biological questions, importance of biological insights, clarity of presentations, expected impact, compatibility with the conference format and, most importantly, correctness, completeness and reproducibility of the studies. After submitting their reviews, the reviewers had the opportunity to discuss the papers and refine the reviews. The ACs facilitated these discussions, oversaw the review process, and made sure the reviews were detailed and consistent with the overall decision. Final acceptance decisions were made in agreement with the SPC.

Throughout the reviewing process, we followed a stringent policy of guarding conflicts of interest. Submissions that had any association to an AC were reassigned to a different area. Care was taken not to assign papers to reviewers with a perceived conflict either (e.g. co-authorship or same institution). The definition of conflict was defined as broadly as possible—including collaborators (present and past few years), same institution (present, past few years, planned future moves), family relations, advisees/advisors, as well as any personal conflicts that could cause the appearance of conflict of interest, or that could genuinely interfere with objective reviewing. Finally, as Proceedings Chairs, we refrained from submitting papers to the conference.

Among the 318 submissions, 58 were accepted for presentation at ISMB 2024 and publication in the Proceedings, conditioned on revisions having properly addressed the comments of the reviewers. In a few cases, the authors had to be reminded to release their source code alongside their manuscript, or clarify some aspects of their evaluations. This year, all conditionally accepted papers were adequately revised and subsequently judged to have properly addressed the concerns of the reviewers. They were thus accepted for the conference Proceedings, resulting in an overall acceptance rate of 18.2%. The acceptance rates for the 11 individual areas are shown in Table 2. Accepted papers were assigned to COSIs based on the preferences of both authors and COSI organizers (Table 1).

We are deeply grateful to the Area Chairs, 375 members of the Program Committee, and 371 subreviewers for their outstanding efforts in conducting thorough and timely reviews. Their contribution is at the core of the scientific quality of the conference. We also thank Bel Hanson, Diane Kovats, and Seth Munholland for their support, guidance, and handling logistical questions, and all the other members of the ISMB Steering Committee for their expert advice and supervision. We also thank the team at Oxford University Press for producing this special Proceedings volume.

Finally, we thank all the authors for submitting their work. These Proceedings would not be possible without the scientific ingenuity of the contributors of all the papers. We recognize that, despite our best efforts, the selection process is necessarily imperfect, and some outstanding work will have been missed. Nonetheless, we hope that all authors received helpful feedback on their work. Finally, we want to thank all the keynote speakers, presenters, and all conference participants.

Thank you all for making this meeting possible, and thus for empowering the entire ISMB community to continue to thrive.

